# An Improved PSO-GWO Algorithm With Chaos and Adaptive Inertial Weight for Robot Path Planning

**DOI:** 10.3389/fnbot.2021.770361

**Published:** 2021-11-05

**Authors:** Xuezhen Cheng, Jiming Li, Caiyun Zheng, Jianhui Zhang, Meng Zhao

**Affiliations:** ^1^College of Electrical Engineering and Automation, Shandong University of Science and Technology, Qingdao, China; ^2^State Grid Dongying District of Dongying City Power Supply Company, Dongying, China

**Keywords:** path planning, improved particle swarm optimization, robot, gray wolf algorithm, adaptive inertia weight, chaos

## Abstract

The traditional particle swarm optimization (PSO) path planning algorithm represents each particle as a path and evolves the particles to find an optimal path. However, there are problems in premature convergence, poor global search ability, and to the ease in which particles fall into the local optimum, which could lead to the failure of fast optimal path obtainment. In order to solve these problems, this paper proposes an improved PSO combined gray wolf optimization (IPSO-GWO) algorithm with chaos and a new adaptive inertial weight. The gray wolf optimizer can sort the particles during evolution to find the particles with optimal fitness value, and lead other particles to search for the position of the particle with the optimal fitness value, which gives the PSO algorithm higher global search capability. The chaos can be used to initialize the speed and position of the particles, which can reduce the prematurity and increase the diversity of the particles. The new adaptive inertial weight is designed to improve the global search capability and convergence speed. In addition, when the algorithm falls into a local optimum, the position of the particle with the historical best fitness can be found through the chaotic sequence, which can randomly replace a particle to make it jump out of the local optimum. The proposed IPSO-GWO algorithm is first tested by function optimization using ten benchmark functions and then applied for optimal robot path planning in a simulated environment. Simulation results show that the proposed IPSO-GWO is able to find an optimal path much faster than traditional PSO-GWO based methods.

## Introduction

Along with the development of automation technology and robotics, path planning is important in robot task execution when searching for an optimal path from the starting position to the target position with obstacle avoidance based on certain criteria.

There have been many achievements in robot path planning. The current path planning algorithms mainly include the colony algorithms (Liu et al., 2019; Ye et al., 2020; Zhang et al., 2020; Zhu et al., 2020), PSO (Krell et al., 2019; Wang Y. B. et al., 2019; Liu X. H. et al., 2021; Song et al., 2021), A^*^ algorithms (Xiong et al., 2020; Liu Z. H. et al., 2021; Tang et al., 2021; Tullu et al., 2021), artificial potential field methods (Wang P. W. et al., 2019; Azmi and Ito, 2020; Song et al., 2020; Yao et al., 2020), genetic algorithms (Hao et al., 2020; Li K. R. et al., 2021; Wen et al., 2021), fuzzy control algorithms (Guo et al., 2020; Zhi and Jiang, 2020), fast marching algorithms (Sun et al., 2021; Wang et al., 2021; Xu et al., 2021), and deep reinforcement learning algorithms (Li L. Y. et al., 2021; Lin et al., 2021; Xie et al., 2021). PSO is an evolutionary computation algorithm that can be used to find the optimal solution through collaboration and information sharing between individuals in the group, as in path planning, the optimal solution is to find the shortest path. The PSO algorithm is easy to implement and has fewer adjustable parameters, however, it still has problems such as being easy to fall into the local optimum and slow convergence.

In response to these problems, researchers have extensively studied PSO improvement in recent years. Das and Jena (2020) used a genetic algorithm that inherits multiple crossover operators and bee colony operators as two evolutionary operators to improve the optimization ability of the PSO. Shao et al. (2020) designed the constant acceleration coefficient and the maximum speed as the adaptive linear variation to adapt to the optimization process. Further, a particle mutation strategy has been proposed to enhance the convergence speed of the algorithm. Li and Chou (2018) applied different strategies to realize the adaptive learning of the PSO; they turned the problem of path planning into a minimizing multi-objective optimization problem and proposed a new adaptive learning mechanism to improve the search ability of the PSO algorithm.

Although the performance of the PSO algorithm has been improved greatly, there are still some shortcomings when it is applied for complex problems (Phung and Ha, 2021), i.e., premature convergence, poor global search capability, and slow convergence speed. To this end, an improved particle swarm optimization combined gray wolf optimization (IPSO-GWO) is proposed in this paper. The GWO can sort the particles during iteration to find the particles with the optimal fitness value and lead other particles to search for the position of the particles with the optimal fitness value, which can greatly improve the search ability of the PSO algorithm in the global searching space. The chaos is further adopted to initialize the speed and position of the swarm particles and a new adaptive inertia weight is designed to improve the global search capability and convergence speed of the IPSO-GWO. When the algorithm falls into the local optimum, chaos can make the algorithm quickly jump out of the local optimum. Experiments on benchmark functions optimization test and the robot path planning simulation tests demonstrate that the IPSO-GWO algorithm has faster convergence speed.

The remainder of the paper is organized as follows. The Proposed Method section describes the proposed algorithm including environment settings, IPSO-GWO, chaos based and new inertial weight design. Experiments and result analysis are explained in third section. The conclusion is given in fourth section.

## The Proposed Method

### Environment Modeling

**Figure 1:**
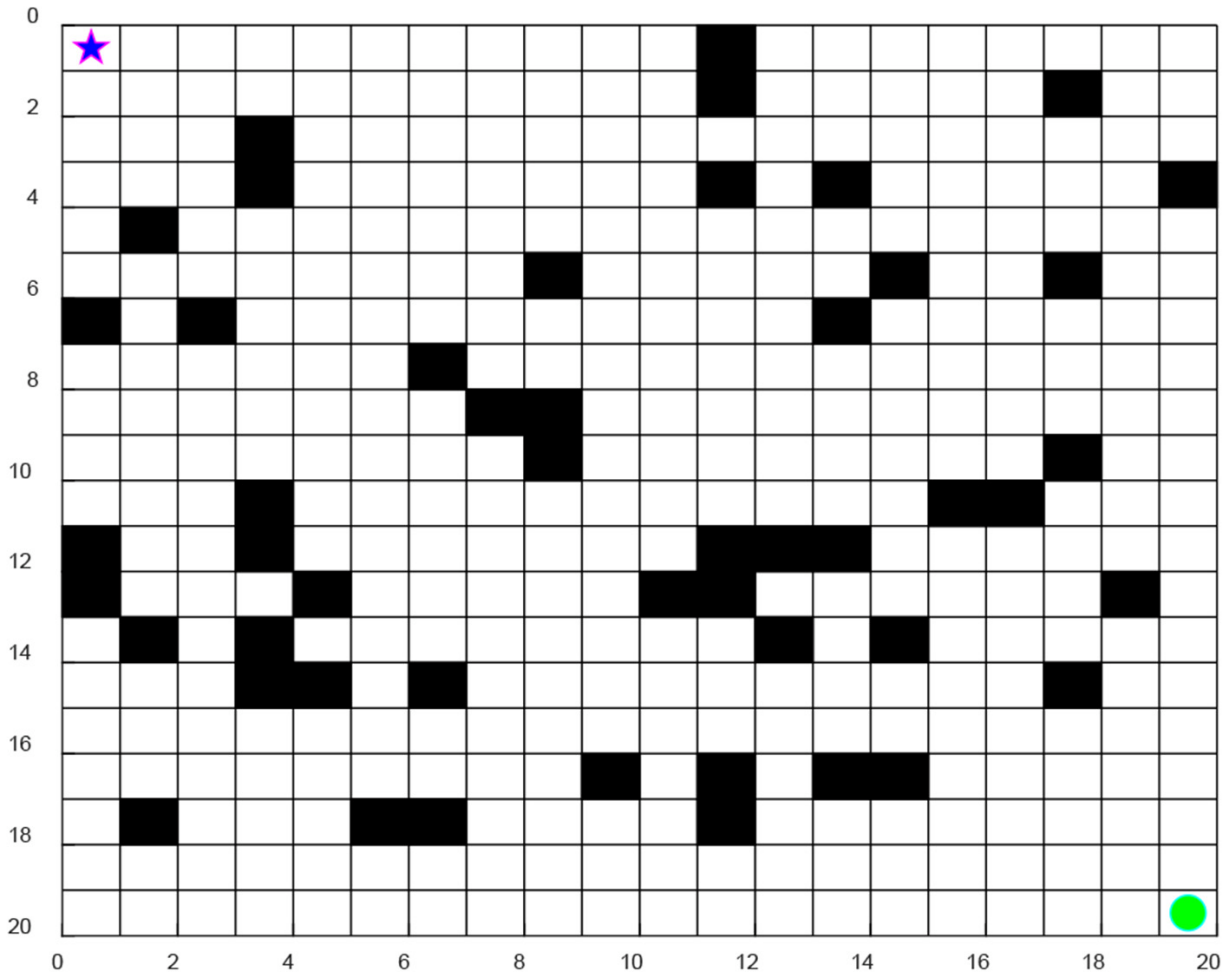
Environment modeling.

The working environment of the robot is established through a grid model, which can be divided into *N* × *N* squares, as seen in Figure 1. The black grid represents obstacles which are impassable, and the white grids represent feasible passing free areas, denoted as 0 and 1, respectively. The five-pointed star indicates the starting point and the green point is the target point. Then the grid model is placed in the coordinate system so as to establish the robot working environment.

It can be seen from Figure 1 that the model is easy to construct, represent, and store data for processing and it is convenient for computer processing.

### PSO Algorithm

The PSO algorithm is an intelligent optimization algorithm proposed by Kennedy and Eberhart (1995) based on the study of the living habits of animal flocks (Tang et al., 2020). Suppose the optimal solution of a certain problem exists in *D* dimensional space for a swarm with size *m*, and the population can be expressed as, *Swarm* = {*x*_1_, *x*_2_, …, *x*_*m*_} where *x*_*i*_(*i* = 1, ⋯, *m*) is the particle without mass, *k* represents the total number of the required iterations, and the position information of the *ith* particle in the *kth* iteration can be represented by a *d*-dimensional vector $x_{i}^{k}=\left(x_{i1}^{k},x_{i2}^{k},\ldots ,x_{id}^{k}\right),i=1,2,\ldots m$, the velocity of each particle can be represented as $v_{i}^{k}=\left(v_{i1}^{k},v_{i2}^{k},\cdots \;,v_{id}^{k}\right)$, *i* = 1, 2, ···, *m*. In each iteration, the position and velocity of the particles are dynamically adjusted according to the historical optimal fitness values of each particle and the population. The calculation for the (*k* + 1)*th* iterations of the *i*th particle in *d*-dimensional space can be written as,


(1) $$\begin{array}{l} v_{id}^{k+1}=v_{id}^{k}+c_{1}*rand()*\left(p_{id}^{k}-x_{id}^{k}\right)+c_{2}*rand()\\ \;*\left(p_{gd}^{k}-x_{id}^{k}\right) \end{array}$$

(2) $$\begin{array}{l} x_{id}^{\left(k+1\right)}=x_{id}^{\left(k\right)}+v_{id}^{\left(k+1\right)} \end{array}$$

where *c*_1_ and *c*_2_ represent the learning factors. *c*_1_ and *c*_2_ are the control variables to control the step lengths of the individual particle flying toward the local optimal value and the swarm optimal value, respectively. $p_{id}^{k}\;$ is the historical optimal fitness value of each particle in the optimization process, $p_{gd}^{k}$ is the optimal fitness value reached by all particles, that is, the optimal fitness value of the population; the *rand*() function is to generate a random number between (0,1) to differentiate particles. The subscript *d*(1 ≤ *d* ≤ *D*) represents the dimension of the searching space. In the above Equation (1) and (2), the speed of the PSO is composed of the local and global three parts: $v_{id}^{k}$ represents the speed of the particle at the *k*^*th*^ iteration, $\left\{c_{1}*rand()*\left(p_{id}^{k}-x_{id}^{k}\right)\right\}$ represents the information of the particle itself, and $\left\{c_{2}*rand()*\left(p_{gd}^{k}-x_{id}^{k}\right)\right\}$ represents the part of the particle in the population for collaboration and information sharing.

### PSO-GWO Algorithm

**Figure 2:**
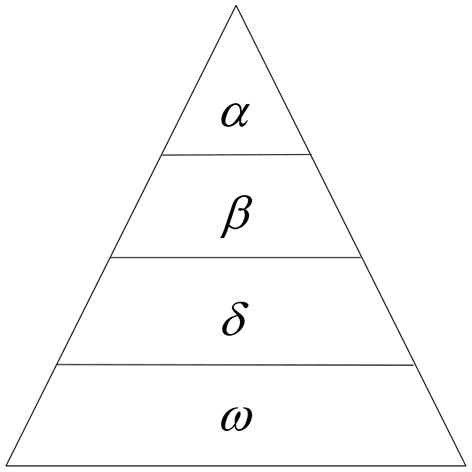
Grey wolf social hierarchy.

The PSO-GWO algorithm is an improved PSO version incentive inspired by gray wolf predation (Narinder and Singh, 2017; Teng et al., 2019; Gul et al., 2021). Different from bird flocks, the gray wolf pack is quite a strict socially hierarchical organization; its hierarchical arrangement is illustrated in Figure 2.

The first layer α in Figure 2 represents the leader in the population, where the leader α is the core of the wolf pack, being mainly responsible for leading and assigning tasks.

The second layer β in Figure 2 represents the think tank team, which is used to assist the leader in management, i.e., when leader α is vacant, β will quickly take over the position of α. In the entire wolf pack, the status of β is only lower than that of α. If α is occupied, β is an advisor to α and discipliner for the group.

The third layer δ follows the command and management of leader α and think tank, and are mainly responsible for care and supervision.

The function of the fourth layer, ω, is to balance the membership within the population.

The essence of the GWO is that the particle with the highest fitness is taken as the leader α to manage other particles. The specific steps of the GWO are summarized as follows:

*Step 1:* To initialize particles of one population in the searching space;
*Step 2:* To rank the particles according to the historical best fitness values;
*Step 3:* Taking three particles with the highest fitness values set as α, β and δ, the other particles are arranged in sequence. If an individual with a higher fitness value appears in the iterative process, it is set as the new leader α and *p*_*gd*_ is updated with its individual fitness. *x*_*i*_ = (*x*_*i*1_, *x*_*i*2_, ..., *x*_*iN*_) represents the position of the *i*th particle, and *v*_*i*_ = (*v*_*i*1_, *v*_*i*2_, ..., *v*_*iN*_) is the speed of the *i*th particle. In the *k* + 1 iteration, the positions of the three particles with the best fitness values in the population are updated *via* Equation (3), and the positions of the rest particles are updated *via* Equations (4) and (5):

(3) $$\begin{array}{l} {\vec{d}}_{\alpha }=|{\vec{c}}_{1}\cdot {\vec{x}}_{\alpha }-w\;*\;\vec{x}|\\ {\vec{d}}_{\beta }=|{\vec{c}}_{2}\cdot {\vec{x}}_{\beta }-w\;*\;\vec{x}|\\ {\vec{d}}_{\delta }=|{\vec{c}}_{2}\cdot {\vec{x}}_{\delta }-w\;*\;\vec{x}| \end{array}$$

(4) $$\begin{array}{l} v_{i}^{k+1}=w*(v_{i}^{k}+c_{1}rand()\left(x_{1}-x_{i}^{k}\right)\\ \;+c_{2}rand()\left(x_{2}-x_{i}^{k}\right)+c_{3}rand()\left(x_{3}-x_{i}^{k}\right)) \end{array}$$

(5) $$\begin{array}{l} x_{i}^{k+1}=x_{i}^{k}+v_{i}^{k+1} \end{array}$$

As seen in Equations (3)–(5), the dimension of the spatial solution is *d*, and the current number of the iteration is *k*. *c*_1_, *c*_2_, and *c*_3_ represent the learning factors, *rand*() is a random number between (0, 1), and *w* represents the inertia weight coefficient. The larger *w* makes the algorithm better in global search, in contrast, the smaller *w* is more suitable for local search. The core idea of the PSO-GWO is to arrange the particles according to their fitness values during each iteration, and set the three particles with the best fitness values to α, β, and δ, while these three particles can predict the approximate range of the location where the optimal solution may exist, and the remaining particles can search the optimal solution within the predicted range. In such a way, the particles can find the optimal solution more quickly and effectively with the improved convergence performance, so the path planning ability of the PSO algorithm can be improved accordingly.

### IPSO-GWO Algorithm

In the previous section, GWO was added to PSO to form the PSO-GWO algorithm, and the search ability of the PSO algorithm can be enhanced to improve its path planning ability, but the PSO algorithm still has the problem of premature convergence, and its convergence speed and global search ability can be further strengthened. Hence chaos and a new adaptive inertia weight are added to provide solutions for these problems.

### PSO With Chaos

The PSO can randomly distribute particles while the optimal solution is highly related to the particle initialization. The more uniform the initial particle distribution, the richer the diversity of the group, and the faster the optimal solution can be obtained.

Chaos (Demir et al., 2020; Lian et al., 2020; Lu et al., 2020; Wu et al., 2020; Guo et al., 2021; Ouertani et al., 2021) refers to a nonlinear motion that can traverse all situations within a specified range. A chaotic sequence can represent all states in a prescribed space, which is commonly generated by mapping. Many researchers have found that chaotic mapping has unpredictable characteristics when studying chaotic mapping relations. Although it is somewhat unpredictable, certain laws can still be used in the mapping process. The most commonly used form of chaotic mapping is logistic mapping, as shown in Equation (6):


(6) $$\begin{array}{l} Z_{i+1}=\mu Z_{i}\left(1-Z_{i}\right)\;i=0,1,2,...;\;\mu \in \left(0,4\right] \end{array}$$

In Equation (6), 0 ≤ *Z*_0_ ≤ 1, *Z*_*i*_ is the value obtained by *i* times Logistic mapping of *Z*_0_, and μ represents the control variable. When μ = 4, the system is within a completely chaotic state and the range of the chaotic space is [0, 1].

The steps of using chaos to initialize the PSO are summarized as follows. First, an *n*-dimensional vector *Z*_1_ = (*z*_1_, *z*_2_, …, *z*_*n*_) is randomly generated, and Equation (6) is used to map the other vectors so as to generate a chaotic sequence *Z*_1_, *Z*_2_, …, *Z*_*N*_. Then the chaotic sequence *z*_*i*_ is inversely mapped from the chaotic space[0, 1] to the space [*a, b*] where the optimal solution is located, and the particle position is *x*_*ij*_ = *a* + (*b* − *a*)*z*_*ij*_, *j* = 1, 2, …, *n*, *i* = 1, 2, …, *N*. Finally, the particles with higher fitness values are determined as the initial particles of the population.

When the PSO is trapped in the local optimum, the algorithm will select the historical optimal value of the particles in the iterative process and convert it into a chaotic sequence through inverse mapping to obtain the optimal position of the particle, then randomly replace a certain particle position in the current search space so that the local optimum can be jumped out by the algorithm. Whether particles fall into precocity is determined by the variance of the population fitness, calculated as,


(7) $$\begin{array}{l} \sigma^{2}=\frac{1}{n}\overset{n}{\underset{i=1}{\sum }}{\left(\frac{f_{i}-f_{avg\;}}{f}\right)}^{2}\\ f=max\left(1,max|f_{i}-f_{avg}|\right) \end{array}$$

where *n* is the size of the population, *f*_*i*_ represents the adaptability of the first particle, and *f*_*avg*_ represents the average adaptability of the current swarm particles. The population variance σ^2^ reflects the precocious state of the particles. When σ^2^ is less than a certain threshold, it is calculated that the particle algorithm will fall into precocity. Then the chaos is applied to process the optimal particles to increase the diversity of the population. The detailed steps are described as follows.

*Step 1:* Select the optimal position in the iterative process and use the function Logistic to map it into the chaotic space [0, 1].
*Step 2:* Use logistics to generate a new sequence and inversely map the sequence to the population.
*Step 3:* Calculate the optimal adaptability of the particles and conclude whether the particle has jumped out of the local optimum; then record the optimal fitness value and set the corresponding particles to α, β, and δ.
*Step 4:* Use the current optimal chaotic particles to manage the particles in the particle swarm to make the particles leave the local optimum.

After the particle swarm performs the chaotic initialization operation, the particles are more evenly distributed in the search space, and the chaotic sequence can be used to reduce the prematurity, improve the diversity of particles, and enhance the convergence speed of the algorithm.

### A New Adaptive Inertial Weight

It is known that the quality of PSO is closely related to inertia weight where the local search ability of the algorithm is higher with smaller inertial weight and global search capability is stronger with larger inertial weight. To enable the algorithm maintaining higher search ability during the entire operation process, many methods have been proposed to adjust the inertia weight (Li et al., 2019a,b; Gopal et al., 2020; Wang et al., 2020; Wang, 2021; Zhang et al., 2021). However, the current inertia weight improvement methods have a close relationship with the iteration number and cannot adapt to the nonlinear variations well. For this reason, this paper deals with the inertia weight *via* the particle adjacent fitness values. The inertia weight can be updated and calculated as,


(8) $$\begin{array}{l} w=\left(w_{max}+w_{min}\right)\;*\;a-\frac{w_{max}\;*\;k}{MaxIter}\\ a=\frac{p_{gbest}^{k}}{p_{gbest}^{k-1}} \end{array}$$

In Equation (8), the global optimal fitness of the *k*th iteration is $p_{gbest}^{k}$, and the global optimal fitness of the (*k* − 1)*th* iteration is $p_{gbest}^{k-1}$; the maximum ω_*max*_ and minimum ω_*min*_ of ω is set as 0.9 and 0.4, respectively. *k* is the current iteration number; *MaxIter* represents the maximum number of the iterations. It can be seen from Equation (8) that *a* is larger at the beginning of the iteration, so the algorithm has strong global searching ability, and *a* becomes smaller gradually at the later iteration stage, so the algorithm has strong local search ability. In summary, the inertia weight combined with the fitness ratio of the neighboring particles can adaptively adjust the size of *w* with the number of iterations so that the algorithm has a higher global search ability.

### Path Planning

The steps of the IPSO-GWO algorithm for path planning are summarized as follows:

*Step 1:* The velocities and positions of the swarm particles are initialized by chaos using logistic function, while the position of each particle represents a path and the fitness of the particle represents the length of the path;
*Step 2:* Collision detection is performed on the path represented by the particles. If the path collides with an obstacle, the path is adjusted without obstacle collision;
*Step 3:* The fitness values of the particles are evaluated to select the three particles with the largest fitness values, set as α, β, and δ;
*Step 4:* To update the positions of particles α, β, and δ based on Equation (3), update the velocities and the positions of the rest particles *via* Equations (4) and (5);
*Step 5:* Determine whether the algorithm has fallen into prematurity *via* Equation (7); if so, chaos is applied to process the premature particles and jump to Step 2;
*Step 6:* Determine whether the algorithm meets the termination condition. If it is satisfied, the iteration stops and the optimal path is obtained; otherwise, continue to Step 2 for calculation.

## Experiment and Result Analysis

### Benchmark Experiments

**Figure 3:**
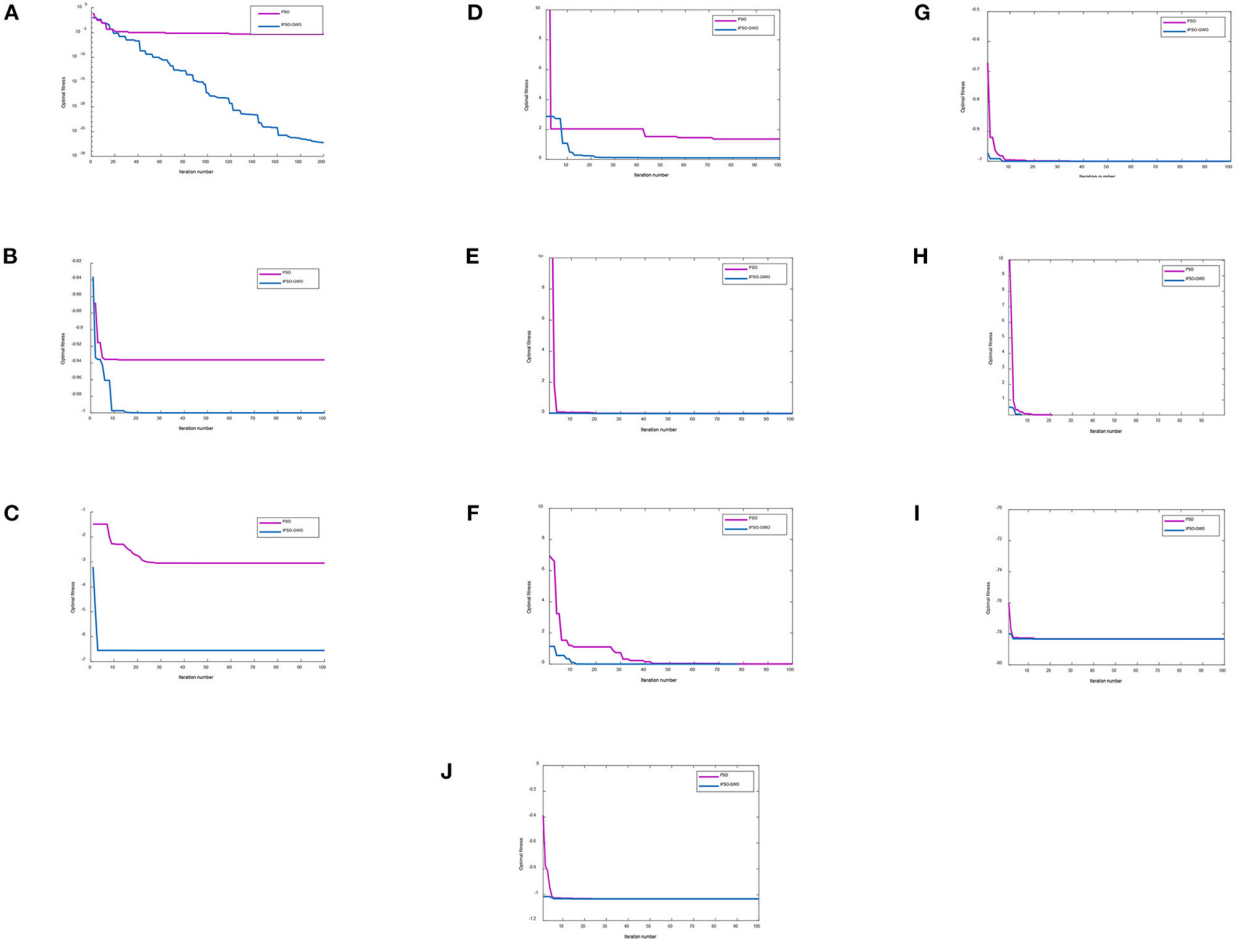
Iteration comparison curves with different functions. **(A)** Iteration curve comparison diagram with Rosenbrock function. **(B)** Iteration curve comparison diagram with Drop Wave function. **(C)** Iteration curve comparison diagram with Peaks function. **(D)** Iteration curve comparison diagram with Bukin function. **(E)** Iteration curve comparison diagram with Booth function. **(F)** Iteration curve comparison diagram with Rastrigin function. **(G)** Iteration curve comparison diagram with Easom function. **(H)** Iteration curve comparison diagram with Levy function. **(I)** Iteration curve comparison diagram with Styblinski-Tang function. **(J)** Iteration curve comparison diagram with Six-Hump Camel function.

To verify the superiority of the IPSO-GWO algorithm, this paper uses MATLAB R2018b software to perform benchmark function tests on PSO and IPSO-GWO. The variables of the simulation experiments are set as follows: the population size is 50, the dimension of the optimization variable is 4, the learning factor *c*_1_ = *c*_2_ = 2.05, and the test functions of the simulation experiments are ten benchmark functions such as Drop Wave, Peaks, Rosenbrock, etc. For Rosenbrock function, the number of the iterations is 200, and the number of iterations of others is 100. Simulation experiments are performed on the above functions. The algorithm iteration curve is shown in Figure 3. The experimental results of the three test functions are analyzed and compared, as listed in Tables 1–10.

**Table 1:** Rosenbrock function test results.

**Algorithm**	**Maximum**	**Minimum**	**Average value**	**Standard deviation**	**Global Minimum**
PSO	1.8143E−12	3.272E−16	1.70543E−13	4.4715E−13	0
IPSO-GWO	9.8215E−27	2.3419E−31	2.2383E−27	2.952E−27	0

**Table 2:** Drop Wavefunction test results.

**Algorithm**	**Maximum**	**Minimum**	**Average value**	**Standard deviation**	**Global minimum**
PSO	−0.93625	−0.99997	−0.99192	0.015633	−1
IPSO-GWO	−0.93625	−1	−0.99636	0.009047	−1

**Table 3:** Peaks function test results.

**Algorithm**	**Maximum**	**Minimum**	**Average value**	**Standard deviation**	**Global minimum**
PSO	−3.0395	−6.5511	−4.62282	1.741408	−6.5511
IPSO-GWO	−3.0496	−6.5511	−5.50012	1.604133	−6.5511

**Table 4:** Bukin function test results.

**Algorithm**	**Maximum**	**Minimum**	**Average value**	**Standard deviation**	**Global minimum**
PSO	27.5233	1.3670	2.1584	3.3801	0
IPSO-GWO	2.8783	0.1078	0.3540	0.7018	0

**Table 5:** Booth function test results.

**Algorithm**	**Maximum**	**Minimum**	**Average value**	**Standard deviation**	**Global minimum**
PSO	16.1673	0.0020	0.3429	2.1682	0
IPSO-GWO	0.0278	0.0001	0.0042	0.0076	0

**Table 6:** Rastrigin function test results.

**Algorithm**	**Maximum**	**Minimum**	**Average value**	**Standard deviation**	**Global minimum**
PSO	6.9599	0.0073	0.5837	1.2599	0
IPSO-GWO	1.1406	0	0.0835	0.2501	0

**Table 7:** Easom function test results.

**Algorithm**	**Maximum**	**Minimum**	**Average value**	**Standard deviation**	**Global minimum**
PSO	−0.6713	−0.9999	−0.9934	0.0351	−1
IPSO-GWO	−0.9727	−1	−0.9992	0.0033	−1

**Table 8:** Levy function test results.

**Algorithm**	**Maximum**	**Minimum**	**Average value**	**Standard deviation**	**Global minimum**
PSO	10.9906	0.0016	0.2517	1.3966	0
IPSO-GWO	0.5333	0.0001	0.0191	0.0890	0

**Table 9:** Styblinski-Tang function test results.

**Algorithm**	**Maximum**	**Minimum**	**Average value**	**Standard deviation**	**Global minimum**
PSO	−76.0206	−78.3296	−78.2937	0.2377	−78.3322
IPSO-GWO	−77.9984	−78.3322	−78.3231	0.0468	−78.3322

**Table 10:** Six-Hump Camel function test results.

**Algorithm**	**Maximum**	**Minimum**	**Average value**	**Standard deviation**	**Global minimum**
PSO	−0.3852	−1.0313	−1.0185	0.0726	−1.0316
IPSO-GWO	−1.0141	−1.0316	−1.0307	0.0035	−1.0316

It can be seen from Figure 3 that, compared with the PSO and PSO-GWO algorithms, the IPSO-GWO algorithm converges the fastest. From Tables 1–10, it is seen that the results obtained from IPSO-GWO is closer to the global minimum, which verify that the performance of the IPSO-GWO algorithm is higher than those of the other algorithms.

### Path Planning Experiments

**Figure 4:**
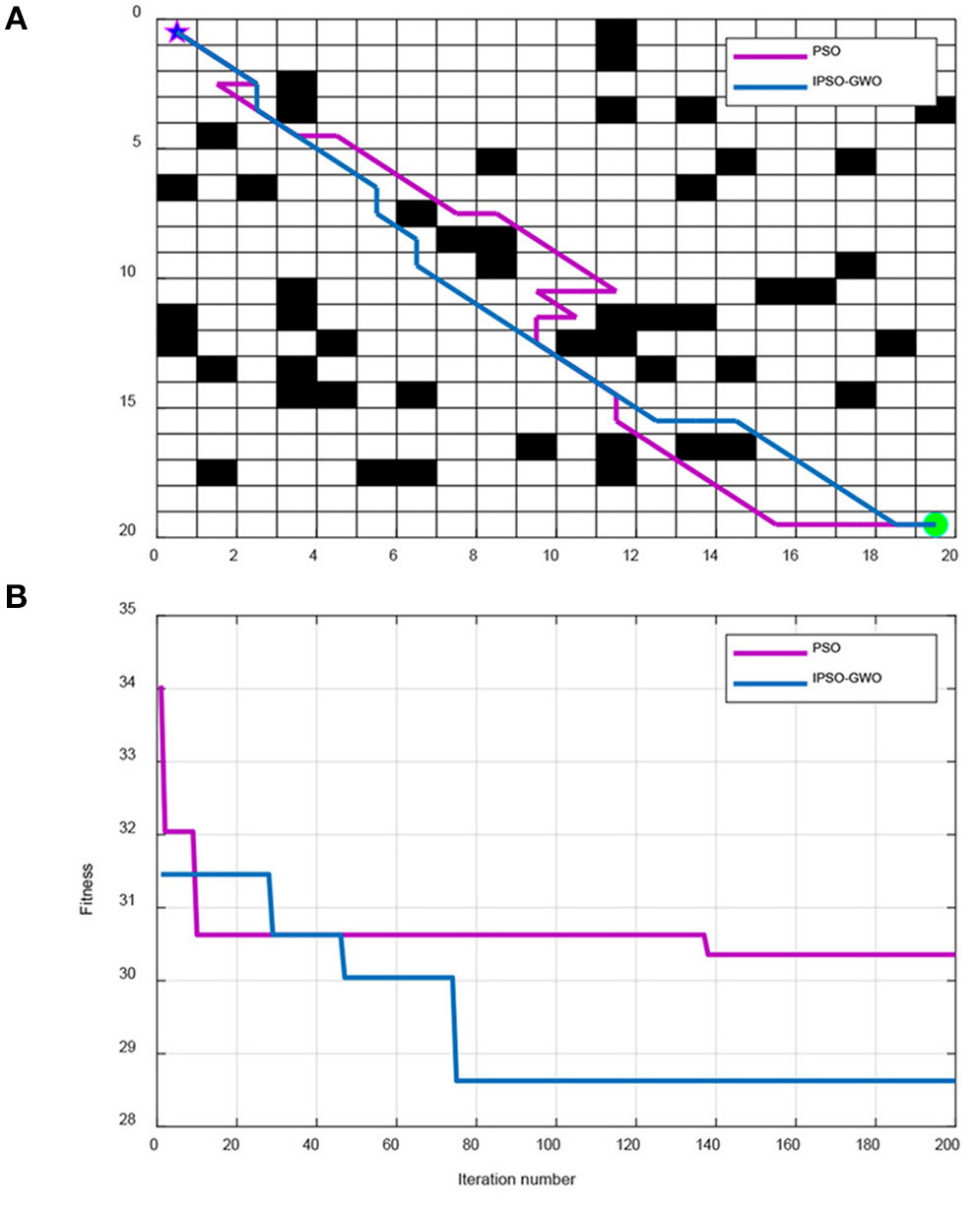
Simulation results of IPSO-GWO and PSO in 20 × 20 map environment. **(A)** Path comparison with PSO and IPSO-GWO algorithms. **(B)** Iterative curves with PSO and IPSO-GWO algorithms.

**Figure 5:**
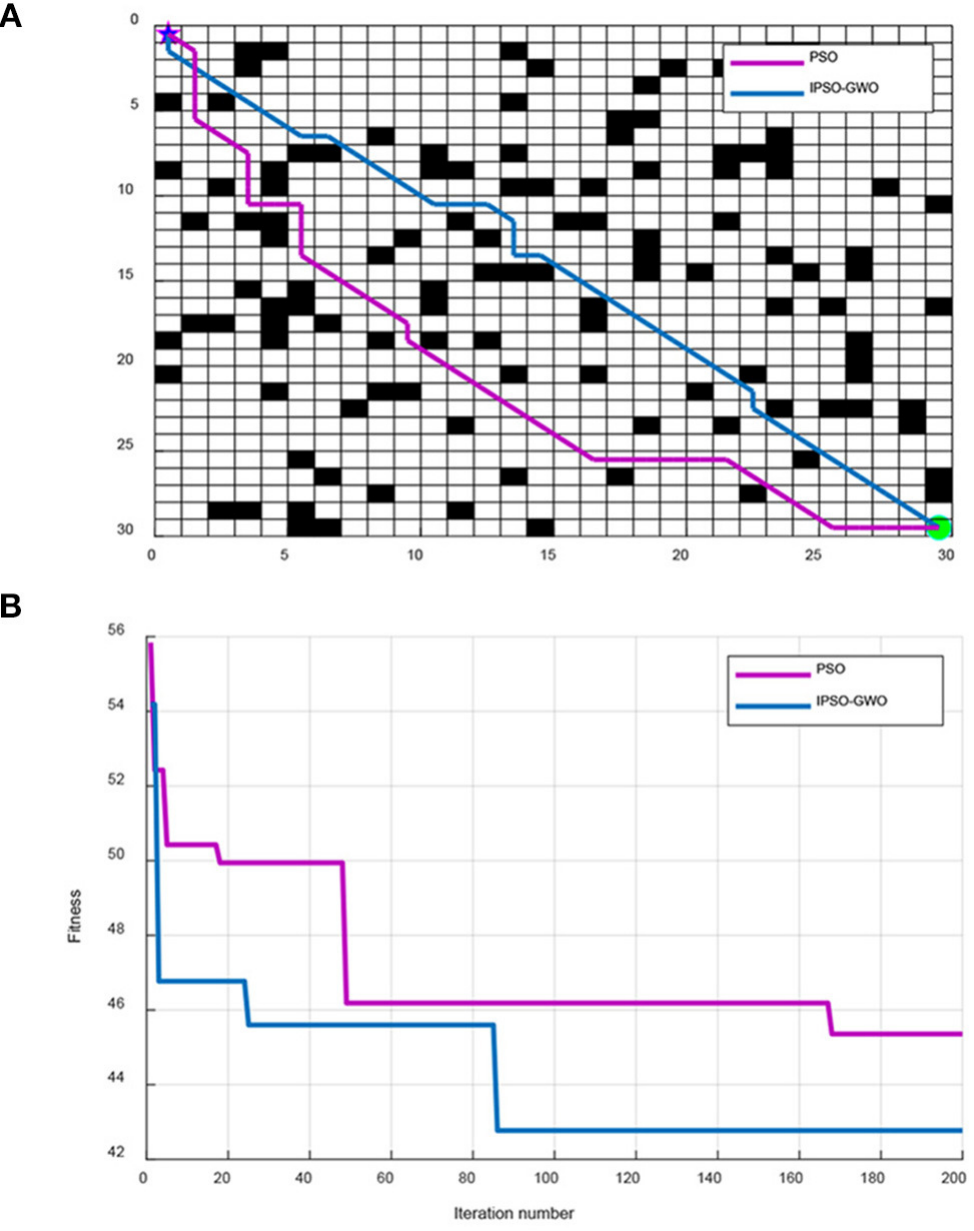
Simulation results of IPSO-GWO and PSO in 30 × 30 map environment. **(A)** Path comparison with PSO and IPSO-GWO algorithms. **(B)** Iterative curves with PSO and IPSO-GWO algorithms.

**Figure 6:**
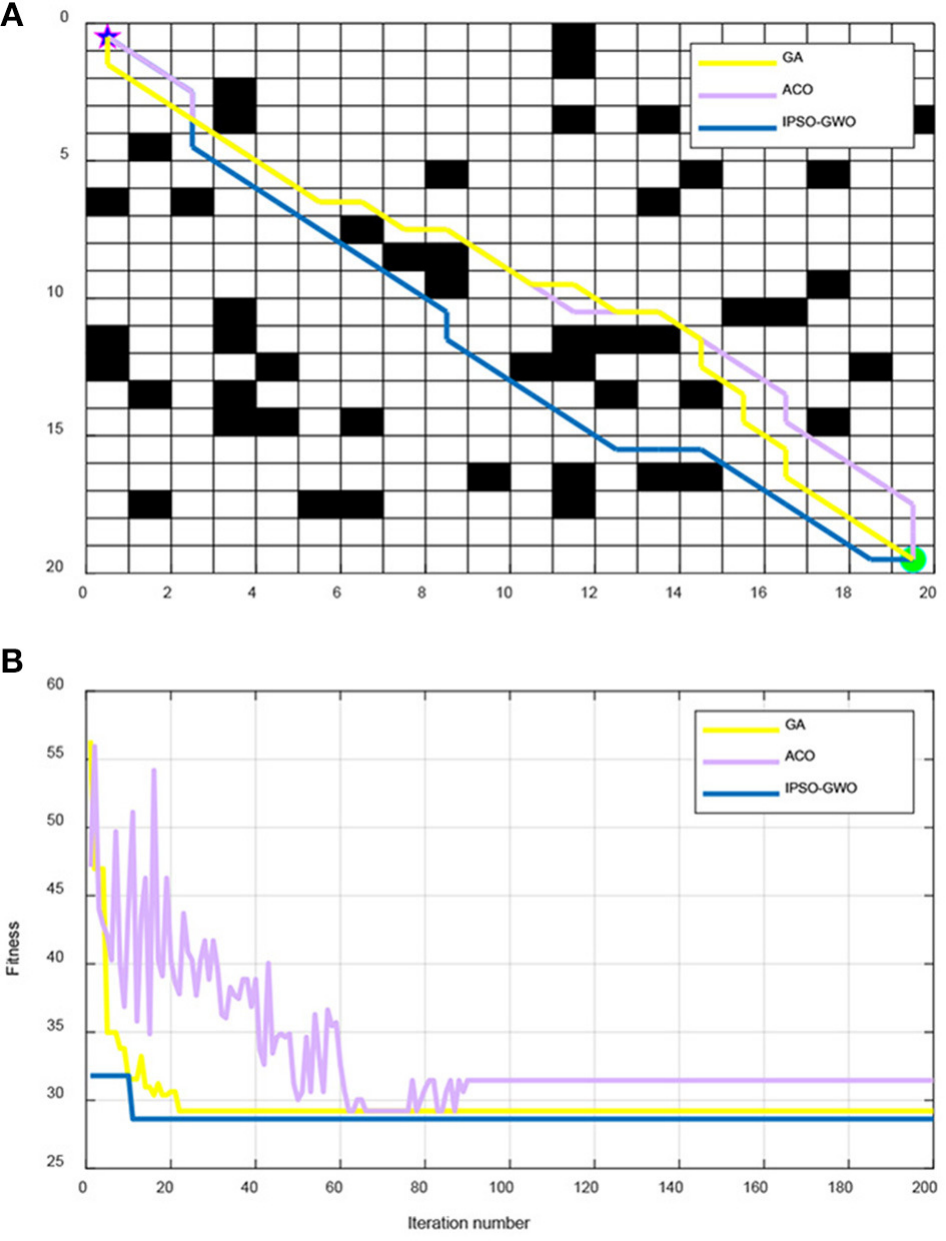
Simulation results of IPSO-GWO, GA, ACO in 20 × 20 map environment. **(A)** Path comparison with GA, ACO, and IPSO-GWO algorithms. **(B)** Iterative curve with GA, ACO, and IPSO-GWO algorithms.

**Figure 7:**
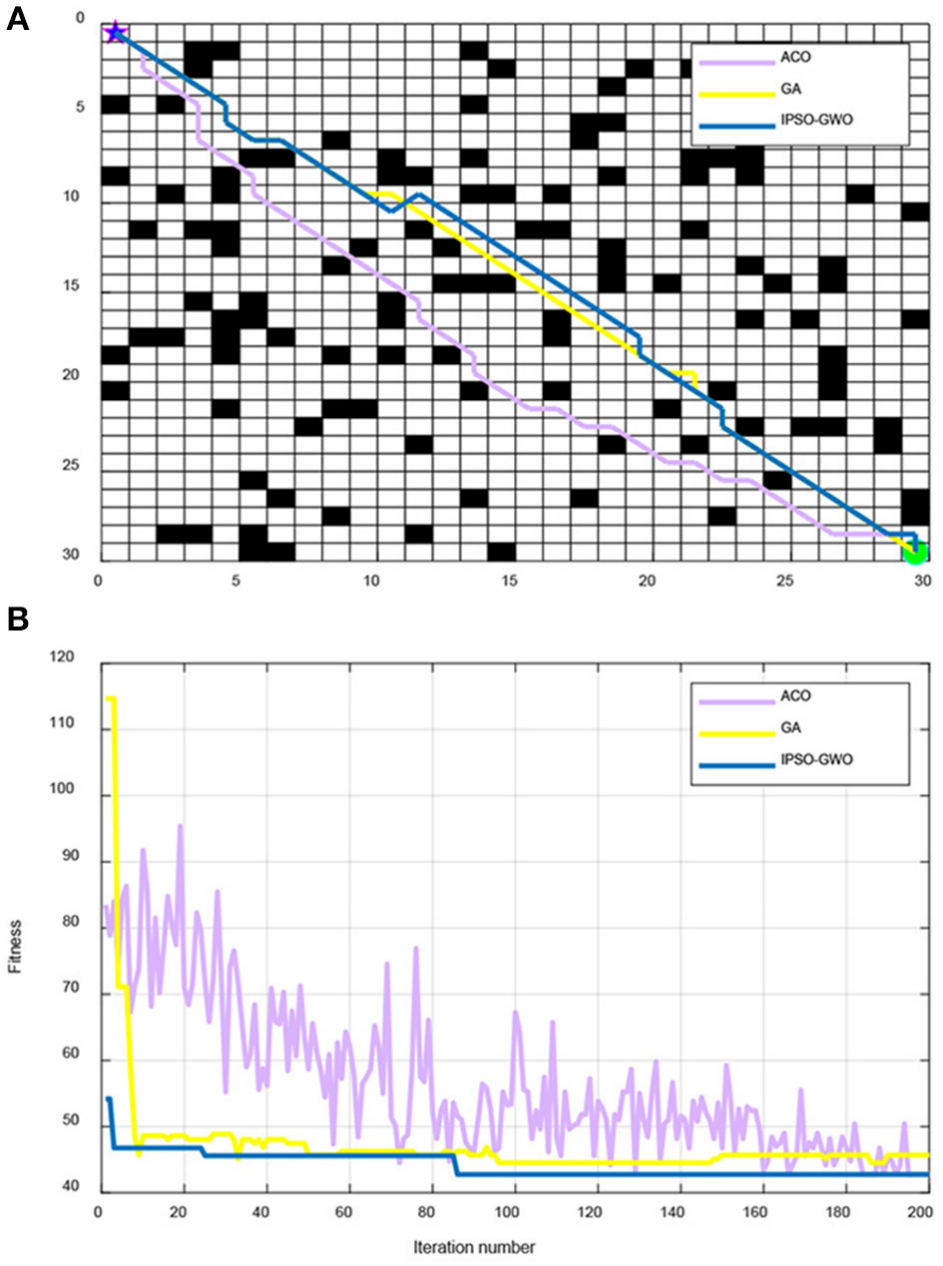
Simulation results of IPSO-GWO, GA, and ACO in 30 × 30 map environment. **(A)** Path comparison with ACO, GA, and IPSO-GWO algorithms. **(B)** Iterative curves with ACO, GA, and IPSO-GWO algorithms.

To verify the superiority of the IPSO-GWO optimization in robot global path planning, we have carried out two kinds of path planning simulation tests: one is the test of IPSO with PSO, and the other is the test of IPSO with Genetic algorithm (GA) and Ant Colony Optimization (ACO). Both tests use 20 × 20 and 30 × 30 map environments. For IPSO-GWO and PSO, the population size is set to 50 and *c*_1_ = *c*_2_= 1.6. For GA, the crossover probability is set to 0.8, the mutation probability is set to 0.2, and the population size is set to 50. For ACO, the stimulating factor of the pheromone concentration α is set to 1, the stimulating factor of visibility β is set to 7, pheromone evaporation coefficient ρ is set to 0.3, pheromone intensity is set to 1, and the number of iterations of the four algorithms is set to 200. The experimental results are illustrated in Figures 4–**7**.

From Figure 4, IPSO-GWO and PSO simulate the path and iterative convergence curve of the path planning in a 20 × 20 map environment; it can be concluded that the PSO can obtain the optimal path at the 138th iteration with path length 30.36. Whereas the proposed IPSO-GWO can acquire the optimization at the 75th iteration with the obtained path length 28.63.

From Figure 5, IPSO and PSO simulate the path and iterative convergence curve of the path planning in a 30 × 30 map environment. The PSO algorithm searches for the optimal path at the 169th iteration, and the obtained path length is 45.36. The proposed IPSO-GWO can find the optimal path in the 86th iteration with acquired path length 42.77.

From Figure 6, IPSO, ACO, and GA are used to simulate the path and iterative convergence curve of the path planning in a 20 × 20 map environment. It can be seen that IPSO-GWO can acquire the optimal path length of 28.63 in the 11th iteration, the optimal path length found by ACO in the 62th iteration is 29.21, and the optimal path length found by GA in the 22nd iteration is 29.21. It can be concluded that the proposed IPSO-GWO algorithm converges faster in a 20 × 20 map environment with shortest path acquirement.

From Figure 7, IPSO, ACO, and GA are used to simulate the path and iterative convergence curve of the path planning in a 30 × 30 map environment. It can be seen that IPSO-GWO can find the optimal path length of 42.77 in the 86th iteration, the optimal path length found by ACO in the 166th iteration is 42.77, and the optimal path length found by GA in the 33rd iteration is 45.11. It can also be concluded that although GA finds the optimal path faster, the path length is longer, whilst ACO finds the same optimal path as IPSO-GWO, but it is slower than IPSO-GWO. In summary, IPSO-GWO algorithm has the highest performance efficiency.

## Conclusion

This paper makes a valuable contribution to the improvement of PSO algorithm in robot path planning in terms of convergence speed and shortest path acquirement. Combining the traditional PSO with the GWO, chaos, and a new adaptive inertia weight, it can address the problem of premature convergence and poor global search ability, and improve the convergence speed for faster path searching. The proposed IPSO-GWO algorithm has been tested against the traditional PSO for ten benchmark functions, and optimization results show that the IPSO-GWO converges faster without premature convergence. Comparing the IPSO-GWO with PSO and two other algorithms for path planning, the IPSO-GWO can find an optimal path with faster speed. In summary, the proposed IPSO-GWO algorithm exhibits higher performance in path planning with the combination of chaos for premature convergence avoidance. In the future, we will continue to apply the proposed IPSO-GWO algorithms in more practical applications.

## Data Availability Statement

The original contributions presented in the study are included in the article/supplementary material, further inquiries can be directed to the corresponding author.

## Author Contributions

XC and JL: proposed the idea and designed the experiment in this study. CZ and JZ: performed the simulation experiments and analyzed the experiment results and wrote the manuscript. MZ: corrected the manuscript. All authors contributed to the article and approved the submitted version.

## Funding

This work was supported by the National Natural Science Foundation of China Program under Grant 62073198, by the Major Research Development Program of Shandong Province of China under Grant 2016GSF117009, and by the China Postdoctoral Science Foundation under Grant 2018M642680.

## Conflict of Interest

The authors declare that the research was conducted in the absence of any commercial or financial relationships that could be construed as a potential conflict of interest.

## Publisher's Note

All claims expressed in this article are solely those of the authors and do not necessarily represent those of their affiliated organizations, or those of the publisher, the editors and the reviewers. Any product that may be evaluated in this article, or claim that may be made by its manufacturer, is not guaranteed or endorsed by the publisher.
